# Surgical treatment of stoma-related hernias: retrospective cohort study of damage claims to the Swedish National Patient Insurance Company 2010–2016

**DOI:** 10.1186/s12893-021-01383-0

**Published:** 2021-11-02

**Authors:** Johan Nyman, Mikael Lindmark, Ulf Gunnarsson, Karin Strigård

**Affiliations:** Department of Surgical and Perioperative Sciences, Surgery, Umeå University, 901 85 Umeå, Sweden

**Keywords:** Parastomal hernia, Stoma-site hernia, Colostomy, Ileostomy, Damage claims

## Abstract

**Background:**

Parastomal hernia and stoma-site hernia are common stoma complications. Parastomal hernia repair is associated with high complication and recurrence rates. Insurance data can provide novel information on the consequences of perioperative complications from the patient’s point of view. The aim was to investigate what types of complications associated with stoma-related hernia surgery that cause patients to apply for economic compensation through the patient insurance system and to investigate demographic and clinical differences among cases based on gender and type of center at which the surgery was performed.

**Methods:**

A national patient damage claim database was searched for ICD-10 codes related to parastomal and stoma-site hernia surgery over a seven-year period. Medical records were screened for claims associated with parastomal hernia repair, relocation or reversal due to parastomal hernia, or stoma-site hernia repair. Claims were classified according to one of four primary complaints: surgical, anesthetic, medical or other. Clinical and demographic differences between genders and hospital types were investigated. Reasons for non-compensation were analyzed.

**Results:**

Thirty claims met the inclusion criteria. Eighteen were related to parastomal hernia repair, seven to stoma-site hernia repair, three to stoma reversal and two to relocation due to parastomal hernia. Twenty-five claims were primarily surgical, two related to anesthesia and three classified as *other*. Seven claims were granted compensation. No demographic or clinical differences were found apart from female gender being associated with previous parastomal hernia repair [6 women and 0 men (*p* = 0.02)].

**Conclusion:**

Surgical complaints predominated. Few claims were compensated, reflecting the complexity and unsatisfactory outcomes of these procedures. Many claims were identified in relation to the incidence of stoma-related hernia surgery.

***Trial registration*:**

Due to its retrospective and descriptive nature, the study was not registered in any registry.

## Introduction

Parastomal hernia (PSH) can be defined as a hernia associated with a stoma [1]. Previous studies using different definitions, diagnostic methods and lengths of follow-up have suggested that more than 30% of stomas will be affected by a PSH within a year of stoma creation, the incidence increasing with time [2]. Approximately 3500 stomas are created annually [3] and approximately 35,000 persons live with a stoma in Sweden [4]. Symptoms of PSH include cosmetic disfiguration and impaired body image, stoma dressing problems leading to leakage and skin irritation, prolapse and life-threatening intestinal incarceration [5]. Indications for surgical treatment commonly include pain, dressing problems, intestinal obstruction or strangulation [6, 7]. Quality of life is reduced among people with a permanent stoma and is further affected by the presence of a PSH [8].

PSH repair is associated with a 13% rate of 30-day reoperation due to postoperative complications and a 6% 30-day mortality rate, emergency PSH repair being an independent risk factor for postoperative complications [9]. Repair is performed with either mesh reinforcement with or without narrowing of the abdominal wall defect using either an open or laparoscopic technique, or suture repair alone; and at present there is insufficient evidence to support preference of any one method, though suture repair, which is associated with a 69% recurrence rate [10], should be avoided at least in the elective setting [2]. It has been suggested that asymptomatic patients or those with comorbidity may be treated conservatively, with relatively low risk for cross-over to surgical treatment or emergency surgery [11]. PSH repair suffers not only from a high recurrence rate, but also complications such as surgical site infection; mesh infection, erosion or adhesion; medical complications; anesthetic problems and death [10].

In a recent nationwide population-based study by our group using a national inpatient register with 99% coverage and scrutinization of the corresponding medical records, the incidence of PSH surgery in Sweden was found to be 71 cases over a nine-year period, extrapolating to 10.5 cases per year when considering missing data [12].

After reversal of a temporary stoma, an incisional hernia at the former stoma site [stoma-site hernia (SSH)] occurs in up to 35% of patients, 50–66% requiring surgical repair [13, 14].

A patient-centered examination of the consequences of stoma-related hernia surgery complications is needed to be able to better understand the morbidity associated with these procedures that have a questionable efficacy and safety. One way of achieving this is to analyze patient insurance data, which includes medical records relevant to the damage claim and the specific case, the complaint filed by the patient, an account of the course of events by the practitioner, the decision (i.e. granted compensation or not) and a report of the reasons behind the decision. This kind of data has been used previously in surgical research. Nordin et al*.* [15] studied claims submitted to the Swedish National Patient Insurance Company (LÖF) related to groin hernia repair (n = 130). Lindmark et al*.* [16] and Ahonen-Siirtola et al*.* [17] studied claims related to ventral hernia repair submitted to LÖF (n = 290) and to the Finnish Patient Insurance Centre (n = 127), respectively. Skogar et al*.* [18] analyzed claims to LÖF regarding bariatric surgery (n = 359). To our knowledge, claims regarding stoma-related hernia surgery have not been investigated.

The primary aim of this study was to explore what types of complications that cause patients to apply for economic compensation through the patient insurance system following stoma-related hernia surgery, to obtain a new patient-centered perspective on stoma-related hernia surgery morbidity. A secondary aim was to investigate differences in demographic and clinical variables based on gender and the type of surgical center at which the surgery was performed.

## Materials and Methods

The LÖF database was scrutinized in January 2018 for claims filed between January 1st, 2010 and December 31st, 2016, using the International Statistical Classification of Diseases and Related Health Problems, 10th revision, Swedish version (ICD-10-SE) [19] codes K43 (Ventral hernia), K45 (Other abdominal hernia) and K46 (Unspecified abdominal hernia). Medical records of the patients filing these potentially eligible claims were then carefully evaluated to assure that only true PSH repair, stoma relocation or reversal due to PSH, and SSH repair were included. Claims were included if any of these procedures were undertaken and excluded if not. Claims were also excluded if the surgical procedure could not be classified from entries in the medical records.

Through medical records and other documents in the LÖF database (such as medical advisor statements, care provider statements and correspondence), detailed information on each case was collected including demographics, hospital type (university hospital or non-university hospital), type of repair, and medical advisor assessment. Claims were classified according to one of four primary complaints: surgical, anesthetic, medical or other. The classification was based on the overall picture, as experienced by the first author. In uncertain cases, consensus between the authors was reached. Analysis of reasons for not receiving compensation was based on the written assessments of the medical advisors to the LÖF. All variables were defined prior to data collection. All data were stored anonymously in a Microsoft Access® (Microsoft Corporation, Redmond, WA, U.S.) database.

The Swedish version of ICD-10, ICD-10-SE, was used to identify claims in the LÖF database registered with the above-mentioned codes. All claims are coded in accordance with the underlying disease for which the patient was treated. Prior to January 1^st^, 2011, an older ICD-10-based classification system was used in Sweden. The relevant codes differ only slightly in their respective descriptions between the two versions, and there were no other codes in the older system that were relevant for inclusion. Prior to data collection, a list of changes in the coding system [20] introduced during the period of the study was assessed to verify consistency.

### LÖF

In Sweden, a patient suffering an injury related to publicly financed healthcare can file a claim to LÖF. Patients are automatically insured by LÖF through the healthcare provider when undergoing medical procedures or participating in medical research. The LÖF claims register covers about 95% of all healthcare provided in Sweden, the remainder comprising private healthcare providers cooperating with their own insurance companies. When a claim has been filed, the LÖF collects relevant material from the patient’s medical records with their informed consent. The consent includes the potential future use of data for research purposes. The LÖF then consults medical advisors from relevant fields of practice to assess the case. If certain criteria are met, according to the Swedish Patient Injury Act [21], the patient may be entitled to compensation. One major criterion is that the complication in question must be avoidable if common practice has been followed. Unavoidable known adverse effects are thus not liable for compensation. Grade of compensation is specified in the Swedish Tort Liability Act [22]. The filing of a claim does not imply a lawsuit against the hospital or the individual practitioner in question, which means that healthcare staff involved in a complication can make statements about the case without risking a lawsuit. Consequently, the process of applying for economic compensation is often initiated and guided by the practitioner involved in the treatment of the patient.

### Statistics

Statistical analyses were conducted in STATA® v.14.2 (StataCorp LLC, College Station, TX, U.S.). Categorical variables were analyzed with χ^2^ or Fischer’s exact test, as appropriate. Continuous variables were analyzed using the independent *t*-test or Mann–Whitney *U*-test, as appropriate. Variables were analyzed according to gender and hospital type (university hospital or non-university hospital). Binary data impossible to extract from medical records or other LÖF documents were entered as *Unknown* in the Access database and then converted to *No* for the statistical analyses. For continuous data, observations were excluded from analysis of the particular variable if data were missing. The sample size was determined by the fixed number of identified cases in the LÖF database.

The study was conducted and reported in accordance with the Declaration of Helsinki and the STROBE statement [23]. The study was approved by the Regional Ethics Committee in Umeå, Sweden (Ref 2017-422-31M), also waiving the need for informed consent due to the retrospective nature of the study.

## Results

**Fig. 1:**
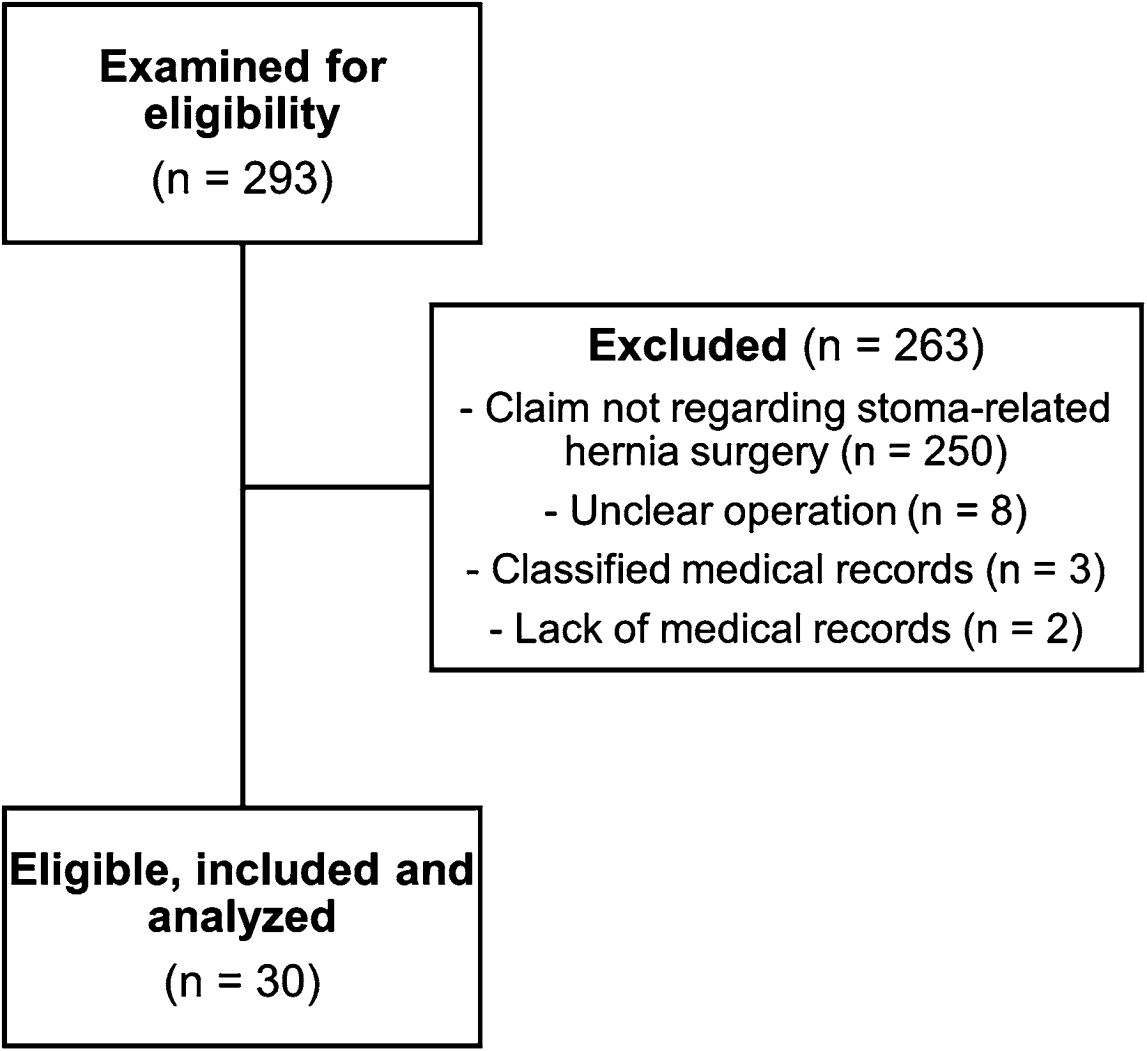
Selection process

The LÖF database search yielded 293 potentially eligible claims. Of these, 30 met the inclusion criteria after scrutiny of medical records. A flow-chart is shown in Fig. 1.

There were 17 women and 13 men. Claims were filed at a median of 160 days after operation (range 13–1100). Eighteen claims were related to PSH repair, seven to SSH repair, three to reversal, and two were related to relocation of stoma due to PSH. Median age at operation was 61 (range 20–81) years. Two claimants had received a prophylactic mesh at the index operation, both eventually claiming compensation for surgical site infection. In one of these cases, manipulation of the mesh caused an accidental enterotomy, whereas a suspected PSH was explored with negative findings in the other. Four operations were emergent. Mesh was applied in 23 cases. Female gender was associated with previous PSH repair (6 women, 0 men, *p* = 0.02). Further demographic and population characteristics are shown in Table 1.

**Table 1:** Demography

	Male	Female	*p*	University hospital	Non-university hospital	*p*
Age, years	61 (20–81)	62 (29–80)	*0.72*	59 (29–80)	62 (20–81)	*0.33*
Smoker	4 (31)	7 (41)	*0.71*	5 (50)	6 (30)	*0.42*
Comorbidity						
Diabetes mellitus	1 (8)	2 (12)	*1.00*	0	3 (15)	*0.53*
COPD	0	2 (12)	*0.20*	1 (10)	1 (5)	*1.00*
CVD	2 (15)	1 (6)	*0.43*	1 (10)	6 (30)	*0.25*
Immunosuppression	2 (15)	1 (6)	*0.57*	2 (20)	1 (5)	*0.25*
Colo- vs. ileostomy			*0.82*			*1.00*
Colostomy	5 (42)	6 (46)		3 (50)	8 (42)	
Ileostomy	7 (58)	7 (54)		3 (50)	11 (58)	
Loop- vs. end ostomy			*0.20*			*0.64*
Loop	5 (42)	2 (15)		1 (17)	6 (32)	
End	7 (58)	7 (54)		5 (83)	13 (68)	
Prophylactic mesh	0	2 (12)	*0.49*	0	2 (10)	*0.54*
Previous PSH repair	0	6 (35)	*0.02*	3 (30)	3 (15)	*0.37*

Demographic characteristics of claimants filing claims to LÖF following stoma-related hernia surgery 2010–2016, stratified by gender and type of operating hospital

Continuous data are expressed as median (range). Categorical data are expressed as numbers

*LÖF* The Swedish National Patient Insurance Company; *COPD* chronic obstructive pulmonary disease; *CVD* cardiovascular disease; *PSH* parastomal hernia

Of the primary complaints, surgical cases (n = 25) were the most common, followed by cases related to anesthesia (n = 2) and other (n = 3); further details are described in Table 2.

**Table 2:** Primary complaints and compensation outcome

	Male	Female	*p*	University hospital	Non-university hospital	*p*
Primary complaint						
Surgical	13 (100)	12 (71)	*0.05*	8 (80)	17 (85)	*1.00*
Anaesthetic	0	2 (12)	*0.49*	0	2 (10)	*0.54*
Medical	0	0		0	0	
Other	0	3 (18)	*0.24*	2 (20)	1 (5)	*0.25*
> 1 complaint	5 (38)	7 (41)	*0.88*	4 (40)	8 (40)	*1.00*
Compensated	3 (23)	4 (24)	*1.00*	4 (40)	3 (15)	*0.18*

Primary complaints and compensation outcome for claims filed with LÖF 2010–2016 following stoma-related hernia surgery, stratified by gender and type of operating hospital

Data are expressed as numbers (%)

*LÖF* The Swedish National Patient Insurance Company

There were no primary medical complaints. Primary complaints filed by men were always surgical. Subtypes of complaints are shown in Table 3.

**Table 3:** Complaint subtypes

Complaint	n
**Surgical**	**25**
Surgical site infection	5
Enterotomy	3
Mesh complication	3
Abscess	2
Fistula	2
Deep infection	1
Bowel obstruction	1
Pain	1
Recurrence	1
Seroma	1
Stoma necrosis	1
Other surgical	4
**Anaesthetic**	**2**
Dental injury	1
Pneumothorax	1
**Other**	**3**

Complaint subtypes in damage claims filed with LÖF 2010–2016 following stoma-related hernia surgery

*LÖF* The Swedish National Patient Insurance Company

Ten claimants underwent surgery at a university hospital (Table 4).

**Table 4:** Operation and postoperative data

	Male	Female	*p*	University hospital	Non-university hospital	*p*
Operation data						
Procedure						
PSH repair-related	7 (54)	11 (65)	*0.55*	7 (70)	11 (55)	*0.69*
SSH repair-related	4 (31)	3 (18)	*0.67*	1 (10)	6 (30)	*0.37*
Relocation^a^ due to PSH	0	2 (12)	*0.49*	1 (10)	1 (5)	*1.00*
Reversal^a^ due to PSH	2 (15)	1 (6)	*0.57*	1 (10)	2 (10)	*1.00*
Emergency operation	1 (8)	3 (18)	*0.61*	2	2	*0.58*
Mesh at current operation	12 (92)	11 (65)	*0.10*	8 (80)	15 (75)	*1.00*
Postoperative data						
Hospital stay, days	15 (3–79)	19 (2–62)	*0.80*	24.5 (4–62)	14.5 (2–79)	*0.40*
ICU admission	4 (31)	4 (24)	*0.70*	3 (30)	5 (25)	*0.77*
ICU stay, days	11 (2–16)	8 (6–15)	*0.72*	15 (6–16)	9.5 (2–11)	*0.29*
Mechanical ventilation	2 (15)	2 (12)	*1.00*	3 (30)	1 (5)	*0.06*
30-day outcomes						
Readmission	4 (40)	8 (50)	*0.70*	4 (44)	8 (47)	*0.90*
Reoperation	5 (42)	10 (63)	*0.27*	6 (67)	9 (47)	*0.34*
Mortality	0	0		0	0	

Operation and postoperative data of cases where damage claims following stoma-related hernia surgery were filed with LÖF 2010–2016, stratified by gender and type of operating hospital

Continuous variables are expressed as median (range). Categorical variables are expressed as numbers (%). The potential presence of additional local repair is demarcated with a

*LÖF* The Swedish National Patient Insurance Company; *PSH* parastomal hernia; *SSH*, stoma-site hernia; *ICU* intensive care unit

Both temporary and permanent stomas were represented, with malignant, benign and emergency surgery indications for the stomas.

Seven (23%) claimants received compensation, there being no significant differences between genders or hospital types. Of the claims leading to compensation, three were related to enterotomy and one each to surgical site infection, recurrence, mesh complication and stoma necrosis.

The remaining 23 cases were not granted compensation. The medical advisors considered the non-compensated iatrogenic injuries unavoidable since common practice was employed (n = 8), or the claim was unrelated to the procedure (n = 2). Infection claims not receiving compensation (n = 10) were deemed not to have been caused by an exogenous infectious agent transmitted at the operation in question. Symptoms were considered to have been adequately interpreted in claims involving delayed diagnosis (n = 3). No claim resulted in the highest degree of compensation (Table 5).

**Table 5:** Compensation outcome

Grade of compensation	n
None	23
Sick leave < 3 months	2
Sick leave ≥ 3 months	2
Invalidity 1–15%	2
Invalidity 16–30%	1
Invalidity > 30%	0
Death	0

Grade of compensation following damage claims regarding stoma-related hernia surgery 2010–2016, according to length of sick leave or degree of invalidity, as judged by the Swedish National Patient Insurance Company. Data are expressed as numbers

*LÖF* The Swedish National Patient Insurance Company

The following variables were infrequently reported in the medical records and hence omitted from analyses: height, weight, body mass index, family history of PSH, American Society of Anesthesiologists class, presence of collagenosis (i.e. Ehlers-Danlos or Marfan syndrome), date of stoma creation, operation time, type of anesthesia, surgical competence and postoperative duration of mechanical ventilation.

## Discussion

In this nationwide study exploring damage claims after stoma-related hernia surgery, surgical complaints predominated (25 of 30 claims). Seven of the 30 claimants received compensation, a lower proportion than in previous reports after surgical procedures. Most non-compensated injuries were considered unavoidable and consequence of the lack of better treatment alternatives.

The low absolute number of observations must be viewed in relation to the overall frequency of stoma-related hernia surgery in Sweden, especially PSH surgery as reported by Odensten [12]. In that study, a mandatory national inpatient register with 99% coverage was searched for diagnosis and procedure codes over a nine-year period. After scrutiny of the medical records of all identified potential cases of PSH surgery, the incidence was found to be on average 10.5 cases per year. Extrapolating from Odensten’s results, the overall incidence of PSH surgery over a seven-year period should be 74 procedures. From this it would appear that approximately 30% of procedures eventually lead to a damage claim (23 of 74). It should, however, be pointed out that data were not available for all surgical centers in Sweden in that study, which implies a theoretical risk of underestimating the total incidence should a high-volume center be omitted. Moreover, it seems reasonable to assume that not all patients suffering a complication file a claim. It is also likely that provision of adequate preoperative information regarding possible risks of the procedure leads to greater acceptance of failure, and therefore a disinclination of patients to file a claim. A greater number of cases included could have perhaps ‘fine-tuned’ the incidence figures a bit, but we doubt that there would be a drastic difference and this was not the primary aim of the study either. The aim was rather to explore what types of complaints that are filed and to what extent compensation was granted. Looking at the types of claims included in this study, there is a quite wide array of different complications and negative experiences, which supports that a good overall view of patient-experienced complications has been reached, despite a low absolute number of cases.

Most complaints being surgical in nature probably arises from the fact that these are technically complicated procedures with unsatisfactory treatment result. The consequences of surgical failure might be more obvious than for medical failure both in terms of impact and relevance to patient insurance. Lindmark et al*.* [16] reported that 5% of claims after ventral hernia repair were medical, and neither Ahonen-Siirtola et al*.* [17] nor Nordin et al*.* [15] explicitly analyzed medical complaints after surgery. Perhaps patients are generally less prone to file a claim regarding a medical complication.

Nordin et al*.* reported 62% and Lindmark et al*.* 40% of claims leading to compensation; figures that reflect the general proportion of claims receiving compensation in the LÖF register. Skogar et al*.* [18] reported roughly the same proportion of claims compensated after bariatric surgery as in the present study (29 vs. 23%), both lower than the overall proportion of compensated claims in the LÖF register. It must, however, be stated that the present study was not bio-statistically designed to explore any difference in compensation between stoma-related hernia surgery patients and the LÖF cases in general or the results of other studies. Nevertheless, we consider the tendency towards lower compensation in this study to be explained by the lack of efficient and safe treatment methods for PSH repair. In a register-based Danish study on PSH repair, Helgstrand et al*.* [9] found 30-day reoperation and mortality rates of 13% and 6% respectively, which shows that surgical treatment of PSH is a high-risk undertaking with poor results in terms of both efficacy and safety. This is highlighted by the fact that the 30-day mortality after emergency resection for colon cancer in Sweden is 6% [24].

The only type of claim that uniformly led to compensation in this study was enterotomy. Inadvertent perforation of bowel is more easily considered avoidable than for example surgical site infection, fistula or recurrence, putting the patient at considerable risk after surgery especially if the indication was debatable in the first place.

Although no previous study has explored reasons for compensation/non-compensation in other forms of hernia repair, it is probable that one reason for lower compensation rates after PSH surgery is that the procedures for groin and ventral hernia repair are technically more mature. Considering the extensive scientific documentation present, the European Hernia Society has presented guidelines with concrete recommendations on inguinal hernia management. Publications on PSH to date do not provide the evidence required for such firm recommendations. Nordin et al*.* reported a similar proportion of surgical claims (78%), which shows that patients subjected to groin hernia repair have roughly the same sort of primary complaint as subjects in the present study. Breakdown of complaints, however, reveals considerable differences. In the study by Skogar et al. only 7% of complaints filed were non-surgical. The discrepancy in compensation rates cannot therefore be solely explained by a difference in primary complaints.

Lindmark et al*.* found 20% of ventral hernia repair claims to be related to anesthesia, a relationship not observed in the present material. Nordin et al*.* found that 11% of claims were anesthesia-related dental injuries. Anesthesia-related complaints accounted for 19% of the procedure-related adverse events in the study by Skogar et al., which in turn accounted for 74% of all claims in that study. Ahonen-Siirtola et al*.* did not specifically analyze anesthesia-related complaints. A summary of the comparative results of these similar studies and the present one is given in Table 6.

**Table 6:** Comparison of types of claims and compensation outcome with similar damage claims studies

	Clinical context	Country	Proportion of surgical claims (%)	Proportion of compensated claims (%)
Lindmark et al*.* 2019	Ventral hernia repair (n = 290)	Sweden	71	40
Nordin et al*.* 2017	Groin hernia repair (n = 130)	Sweden	78	62
Ahonen-Siirtola et al*.* 2015	Ventral hernia repair (n = 127)	Finland	a	38
Skogar et al. 2015	Bariatric surgery (n = 359)	Sweden	93^b^	29
Nyman et al	Stoma-related hernia surgery (n = 30)	Sweden	83	23

^a^Only surgical complaints analyzed

^b^‘Procedure-related AEs’ and ‘Late AEs’ combined

Despite the relatively high number of cases included in relation to the assumed incidence of stoma-related hernia surgery in Sweden, this study is limited in the absolute number of observations. Another limitation is that LÖF data were not matched with a national register regarding incidences of the respective procedures and associated complications not represented in the LÖF database during the study period. The Swedish National Patient Register (NPR) holds patient-related, geographical, administrative and medical data (such as diagnosis and procedure codes) for inpatient care but does not include corresponding medical records. The rationale behind not collecting data from the NPR was the risk of including procedures that we did not intend to study, mainly primary or incisional ventral hernia repair. In the present study, 250 of 293 potentially eligible cases from the initial database search were excluded after scrutiny of medical records due to the claims not being related to relevant procedures, showing that any incidence figure calculated from NPR data would have been virtually impossible to interpret. If a design with matching to NPR data were to be used, it would be necessary to collect operative notes for all the cases, which in practical terms would imply asking every surgical clinic in Sweden to send these files. Such a method would probably result in less trustworthy results due to missing data, as not all centers would be expected to attend such a procedure. In the study by Odensten et al*.* it was not possible to cover all surgical centers for that reason. The LÖF data source, on the other hand, has national coverage so even if calculations regarding incidences of complications should be interpreted with caution, the tendency regarding types of complaints and reimbursement outcomes is representative. It should also be kept in mind that damage claim analyses represent a selected population of patients who are not only affected by a complication but also has been informed about the possibility of applying for economic compensation and either have the willpower and resources to file a claim themselves or have adequate support from relatives or healthcare staff to do so. Another limitation is that important outcome measures were subjected to case-to-case judgements by the authors. In addition, there was a probable lack of statistical power for secondary outcome measures and generalizability might be limited by legislative differences between countries.

A strength of this study is its population-based data covering virtually all procedures performed in Sweden (10 million inhabitants). The study was not intended to make a bio-statistical evaluation of the outcomes of stoma-related hernia-surgery, but rather to give a description of the consequences of its complications and thus adds a different perspective, given that most previous research has focused on outcomes such as recurrence, surgical site infection and reoperation. To achieve the individual patient’s experience of complications, damage claim data is used instead of focusing on outcomes defined beforehand by the researchers as is the case for forms or reviews of only the medical records. Other strengths of the study are that this is the first study to assess patient damage claims associated with stoma-related hernia surgery alone and its less selected demography with, for instance, paraileostomy and paracolostomy hernias, inclusion of SSHs and that different techniques for PSH repair are represented. Furthermore, reasons for non-compensation are analyzed.

## Conclusions

Our findings suggest that a high proportion of patients undergoing stoma-related hernia surgery eventually file a patient damage claim, and that surgical complications predominate, which emphasizes the need for safer treatment methods, or ideally effective and safe primary prevention. Compensation was rare. Further research using patient damage claims should focus on reasons for refraining from filing a claim by patients suffering from a complication, as well as observations over a longer period of time.

## Data Availability

Anonymised datasets used and analysed during the current study are available from the corresponding author on reasonable request.

## Declarations

### Methods and reporting guidelines statement

The study was conducted and reported in accordance with the Declaration of Helsinki and the STROBE statement for observational studies as well as national research regulations.
